# Construction of Biocompatible Hydrogel Scaffolds With a Long-Term Drug Release for Facilitating Cartilage Repair

**DOI:** 10.3389/fphar.2022.922032

**Published:** 2022-06-16

**Authors:** Wei Zhang, Rui Chen, Xiong Xu, Liang Zhu, Yanbin Liu, XiaoJie Yu, GuoKe Tang

**Affiliations:** ^1^ Joint Surgery Department, Zhuzhou Central Hospital, Zhuzhou, China; ^2^ Department of Orthopedics, Second Affiliated Hospital of Naval Medical University, Shanghai, China; ^3^ Department of Graduate, Hebei North University, Zhangjiakou, China; ^4^ Department of Orthopedics, Shanghai General Hospital, Shanghai Jiaotong University, Shanghai, China; ^5^ Department of Orthopedics, Hunan Aerospace Hospital, Changsha, China

**Keywords:** BMSCs, cartilage regeneration, hydrogel scaffold, KGN, long-term release

## Abstract

In tissue engineering, hydrogel scaffolds allow various cells to be cultured and grown *in vitro* and then implanted to repair or replace the damaged areas. Here in this work, kartogenin (KGN), an effectively chondro-inductive non-protein bioactive drug molecule, was incorporated into a composite hydrogel comprising the positively charged chitosan (CS) and methacrylated gelatin (GelMA) polymers to fabricate appropriate microenvironments of bone marrow mesenchymal stem cells (BMSCs) for cartilage regeneration. Based on the combination of physical chain entanglements and chemical crosslinking effects, the resultant GelMA-CS@KGN composite hydrogels possessed favorable network pores and mechanical strength. *In vitro* cytotoxicity showed the excellent biocompatibility for facilitating the cell growth, adhesion, proliferation, and differentiation. The long-term sustainable KGN release from the hydrogel scaffolds *in situ* promoted the chondrogenic differentiation that can be employed as an alternative candidate for cartilage tissue regeneration.

## Introduction

The intrinsic properties like avascularity and low cellularity of articular cartilage have restricted its spontaneous regeneration for many years. The occurred cartilage defect always gradually gives rise to the degeneration of entire joint, thereby resulting in the severe osteoarthritis and appealing for invasive intervenes (Klein, 2009; Huey et al., 2012; Anetzberger et al., 2014; Makris et al., 2015; Li et al., 2017). To prevent the progression of aggressive lesion on the articular cartilage, surgical interventions are desperately required and updated. In this cause, several clinical managements are performed for cartilage repair in recent years, including the microfracture, mosaicplasty, and autologous chondrocyte implantation. Nevertheless, all these current clinical strategies are controversial because of the limited transplantation materials and dissatisfied long-term rehabilitation, thus necessitating the development of alternative designs and strategies (Detterline et al., 2005; Marlovits et al., 2006; Li et al., 2015; Mardones et al., 2015; Yan et al., 2020). Fortunately, the advent and development of tissue engineering and controlled drug release techniques provide the promising therapeutic for cartilage reconstruction, wherein the engineered scaffold is recognized as a crucial ingredient for cell activity, proliferation, and differentiation (Tang et al., 2020; Wang et al., 2020; Xu et al., 2020; Hua et al., 2021; Xu et al., 2021).

Nowadays, various tissue engineered scaffolds have been fabricated for the cartilage regeneration, including natural or synthetic polymer hydrogels with three-dimensional (3D) porous architectures by the chemical or physical cross-linker (Langer and Vacanti, 1993; Das et al., 2015; You et al., 2019; Dou et al., 2020; Zhu T. et al., 2022). Naturally derived hydrogels are particularly appealing because of their inherent biocompatibility, biodegradability, and biosafety (Burdick and Prestwich, 2011; Seliktar, 2012; Li et al., 2016; Stratton et al., 2016). As the representative materials, gelatin derived from the hydrolysis of collagen possessed the extremely similar component of ECM and cell-friendly essence, while the sole alkaline polysaccharide of chitosan (CS) displayed distinct biochemical characteristics to promote the cell growth, adhesion, proliferation, and differentiation. It was found that CS-mediated expression of chondrocytes phenotype was favorable for the tissue repair in the regenerative medicine. Despite the progress achieved in the development of hydrogels over the past few decades, the poor strength and low biological activity still limited their clinical applications. Therefore, pursuit of effective physical interaction with tailored architectures and functions and the appropriate incorporation of bioactive therapeutics into hydrogels to promote cartilage regeneration are promising strategies on facilitating the therapeutic efficacy of cartilage regeneration.

Kartogenin (KGN) as a non-protein small molecule chondrogenesis-inducing agent is reported to significantly promote chondrocyte differentiation capacity in a dose-dependent manner with excellent safety. Compared to the short half-life of biological protein and/or growth factors, KGN is capable of maintaining the biological activity and stability for exerting its long-term chondrogenesis on tissue regeneration (Johnson et al., 2012; Xu et al., 2015; Yuan et al., 2021). By means of this unique advantage, development of a suitable KGN delivery system to maintain the effective drug concentration at the site of cartilage defect is essentially significant for the cartilage tissue repair. Here in this work, we designed and prepared a KGN-encapsulated GelMA-CS composite hydrogel to support BMSCs for effective cartilage regeneration. GelMA-CS hydrogel possessed suitable network pores, good mechanical strength, and excellent biocompatibility that associated with the extracellular matrix for facilitating the cell viability, adhesion, growth, and proliferation. Owing to the electrostatic interactions between the oppositely charged groups, the therapeutic drug of carboxyl modified KGN could be efficiently encapsulated and absorbed within the amine-abundant hydrogel networks, demonstrating a locally sustained delivery and elucidating therapeutic efficiency on chondrogenic differentiation and cartilage regeneration. Thereafter, these therapeutic agent-loaded GelMA-CS@KGN hydrogel scaffolds were demonstrated to exhibit great clinical potential in cartilage regeneration in the future.

## Materials and Methods

### Materials

Gelatin (80–100 kDa, J & K), methacrylic anhydride (94%, J & K), and CS (degree of deacetylation >90%, viscosity: 45 mPa s) were obtained from Shandong Jinhu Co., Ltd. Lithium phenyl-2,4,6-trimethylbenzoylphosphinate (LAP, J & K) and kartogenin were obtained from KGN, Selleckchem Co., Ltd. The cholecystokinin octapeptide kit was obtained from Beyotime Co., Ltd. All other reagents were purchased from Sigma-Aldrich and used as received without further purification. Cells were supplied by China Infrastructure of Cell Line Resource.

### Preparation of Methacrylated Gelatin–Chitosan and Methacrylated Gelatin–Chitosan@Kartogenin Composite Hydrogels

GelMA was synthesized according to the previous literature (Zhu W. et al., 2022). The GelMA-CS composite hydrogel was simply prepared by adding CS solution (3 wt%) into the GelMA solution (20 wt%), containing the photoinitiator of LAP molecule. The KGN-loaded DN hydrogel was obtained by mixing the 100.0 μg/ml of KGN into the molds under UV irradiation at room temperature.

### Morphology and Pore Size of Composite Hydrogels

Scanning electron microscopy (SEM) was employed to observe the microstructure of hydrogels under at acceleration voltage of 5 kV on a JSM-6700F microscope. The freeze-dried samples were sputter coated with a thin layer of Pt for 90 s to make the samples conductive before testing.

### Rheology of Composite Hydrogels

Rheology was carried out on a rheometer (Thermo Haake Rheometer, United States). The hydrogels were spread on a parallel plate (25 mm) and sealed with silicone oil to prevent the solvent evaporation. A dynamic frequency scan in the range from 0.1 to 100 rad/s was used to record the storage and loss moduli G′ and G′′. The samples were measured with the stress amplitude and temperature of 0.1% and 25°C, respectively.

### Compressive Behavior of Composite Hydrogels

The compressive measurement of hydrogels was calculated with a universal material testing machine of Instron 3365 (Instron Co., United States). The samples were cut into cylinders (diameter = 15 mm and height = 7.5 mm), and compressive measurement was carried out at a rate of 1 mm/min.

### Kartogenin Release From Composite Hydrogels *In Vitro*

The hydrogel was prepared in a container with the diameter of 15 mm and height of 7.5 mm, and 100.0 μg/ml of KGN drug was encapsulated inside the hydrogel, which was immersed into the PBS solutions, and then collected at the predetermined intervals using the UV–vis spectrophotometry with the absorption peak of 278.4 nm.

### Cytotoxicity

Cytotoxicity of hydrogel was measured using Cell Counting Kit-8 assay by contacting the hydrogel extracts. Bone marrow mesenchyml stem cells (MSCs) were seeded and incubated at 37°C in 5% CO_2_ for 12 h, and then hydrogel extracts were added into each well for further predetermined incubation time. After incubation for predetermined time, the cell culture medium was removed and 100 µl of fresh culture medium and 10 µl of CCK-8 were added to the 96 wells. Subsequently, cell viability was evaluated by comparing the absorbance of measured solutions using micro-plate reader at 450 nm. The cells cultured in DMEM medium containing 10% FBS were used as controls. The final results were assumed to be the means of triplicate. Cell viability (%) was calculated by the following equation:

$$\text{Cell viability }\left(\text{\%}\right)=\left({\text{A}}_{\text{sample}}-{\text{A}}_{\text{blank}}\right)/\left({\text{A}}_{\text{control}}-{\text{A}}_{\text{blank}}\right)\times 100\text{\%}\text{.}$$

### Live/Dead Staining Assay

Cell live and dead viability was determined by live/dead viability according to the manufacturer’s instruction. After cultivating for prescribed time intervals, BMSCs were washed by cold buffer solution for three times and immersed with 4% paraformaldehyde solution for 30 min at room temperature to fix the cell configuration. Then, fluorescent propidium iodide (PI, red) stain and fluorescent (SYTO 9, green) stain were added to the reaction mixture and incubated at room temperature for 20 min. Finally, these BMSCs were washed by buffer for three times and directly transferred to glass culture dish for further confocal laser scanning microscopy (CLSM) observation.

### Quantification of DNA, GAG, and COL-2 Content

ELISA was used to quantitatively evaluate the extracellular protein produced by the BMSCs in hydrogels. The COL-2 content was tested by an ELISA kit following the manufacturer’s instructions. The DNA content was detected using a fluorometric assay. Aliquots of sample digestion were stained with 200 μl of Hoechst 33258 solution at 37°C for 20 min, followed by the measurement with an excitation wavelength of 360 nm and an emission wavelength of 460 nm. The DNA content was normalized with a standard curve of calf thymus DNA. In addition, the content of proteoglycan was detected from the GAG content by the 1, 9-dimethyl methylene blue dye-binding assay. Total GAG was normalized to the total DNA content by adding 20 μl of sample into 200 μl of DMMB solution with the measurement absorbance at 525 nm.

### Semiquantitative RT-PCR

The TRIzol reagent was used to extract the total RNA when the cell-hydrogel composites were incubated for 7 and 14 days. The RNA was reversely transcribed into cDNA by using MMLV reverse kit, and RT-PCR was conducted by the real-time PCR system with SYBR Green PCR Master Mix.

### Statistics Analysis

All statistical results were expressed as mean ± standard deviation of more than three times for each experiment. The differences among groups were calculated by one-way analysis of variance (ANOVA) after testing for homogeneity of variances, *p* < 0.05 was considered statistically significant.

## Results and Discussion

### Fabrication and Characterizations of Methacrylated Gelatin–Chitosan and Methacrylated Gelatin–Chitosan@Kartogenin Composite Hydrogels

**FIGURE 1:**
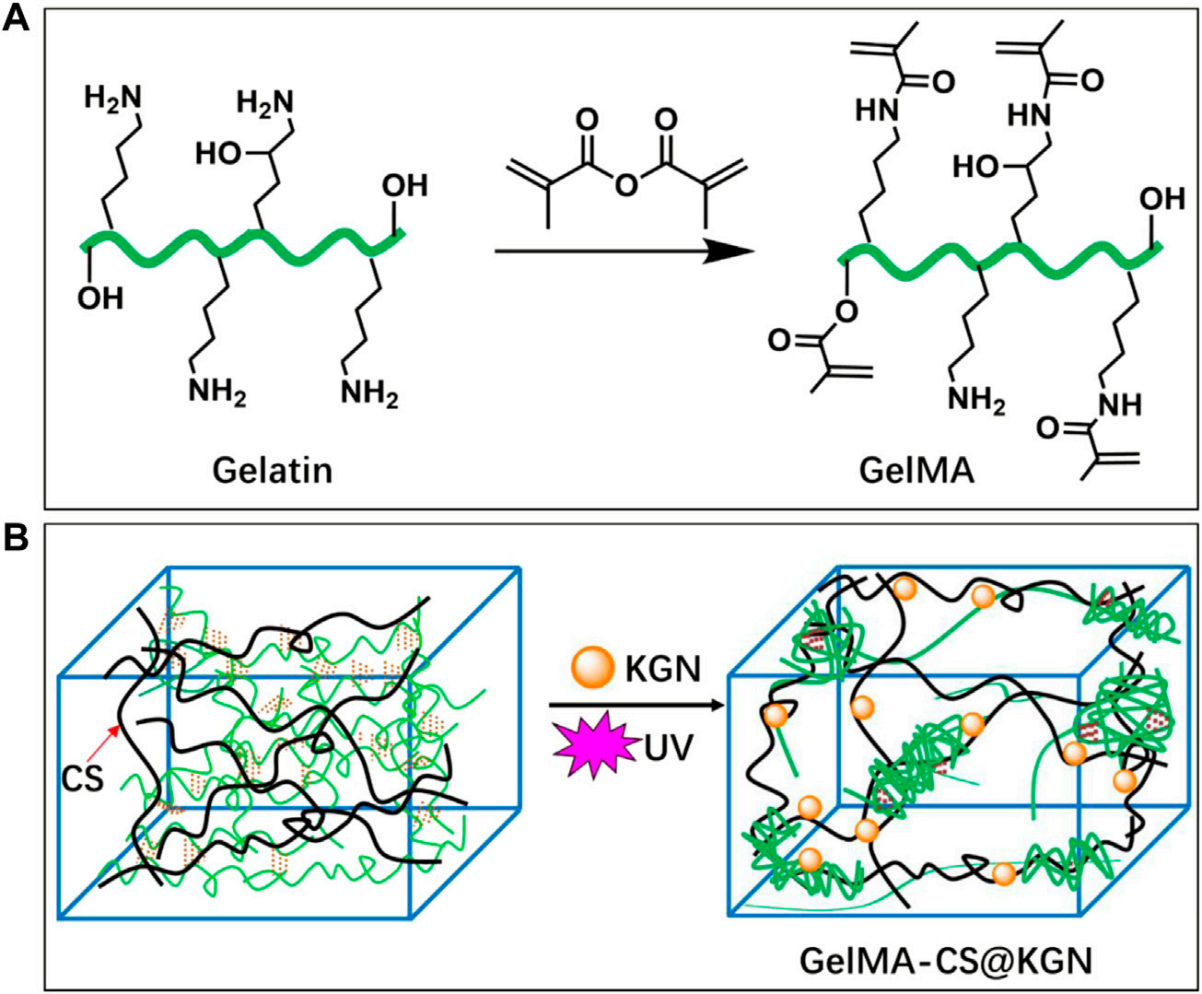
Schematic illustrations of synthesis pathways of **(A)** GelMA macromolecule and **(B)** KGN-loaded GelMA-CS@KGN composite hydrogel.

The preparation route of GelMA-CS@KGN composite hydrogel is observed in Figure 1. The UV irradiation could make the quick formation of the crosslinking gelatin network at the absence of the photoinitiator. However, its poor mechanical property required the incorporation of other polymers or components. Therefore, a non-covalent robust CS network was incorporated by the physically electrostatic interactions and chain entanglement effects between these two natural polymeric chains to enhance the mechanical strength. During this process, the therapeutic KGN agents could be *in situ* encapsulated by the electrostatic interactions among the -NH_2_ groups of GelMA and CS and -COOH group of KGN. Under this circumstance, the KGN molecules could be slowly released from the composite hydrogels for a long time.

**FIGURE 2:**
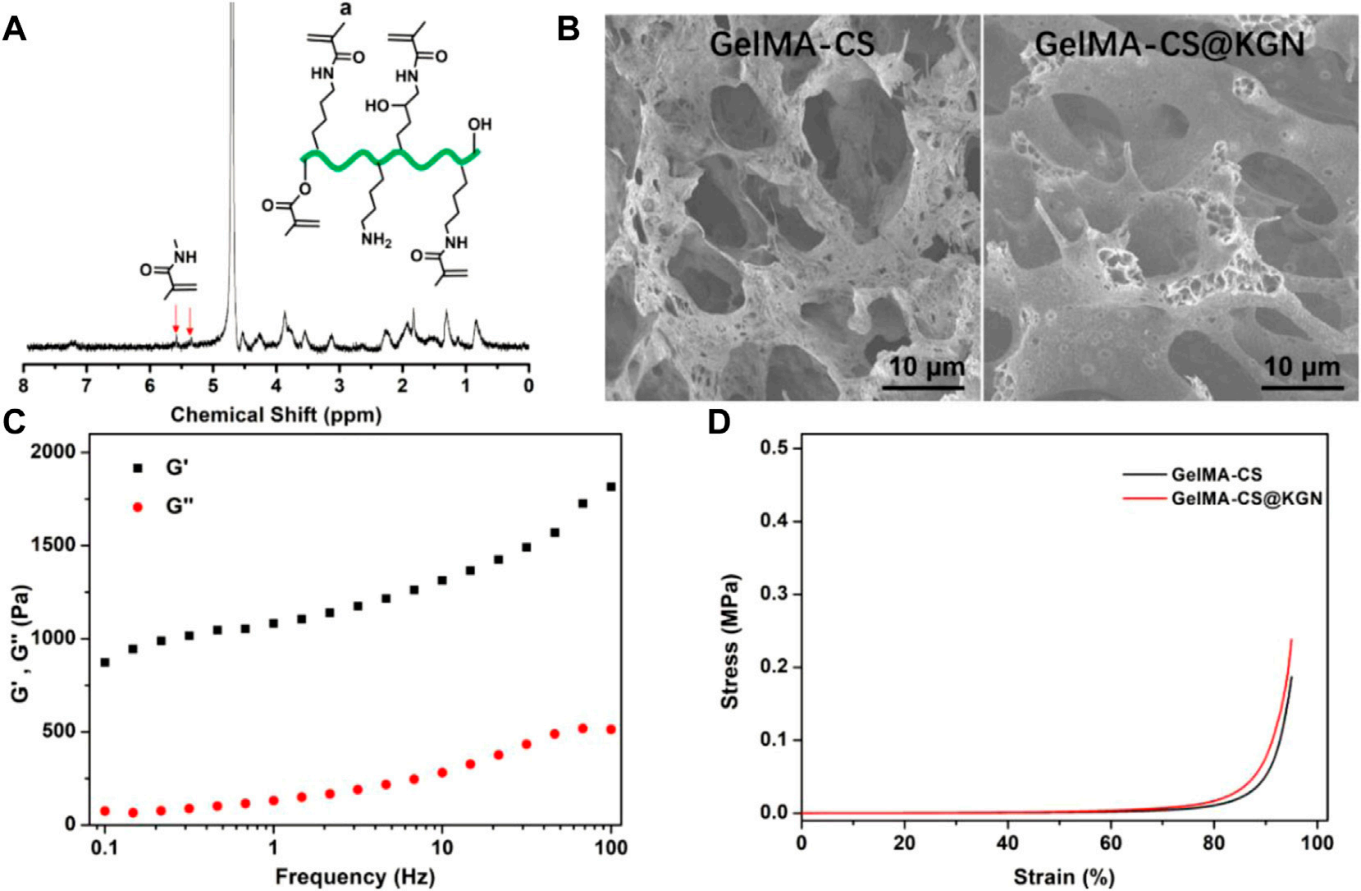
**(A)** ^1^H NMR spectrum of GelMA. **(B)** SEM images of GelMA-CS and GelMA-CS@KGN hydrogels. **(C)** Rheology of the GelMA-CS. **(D)** Compressive curves of GelMA-CS hydrogels with or without KGN laden.

As shown in Figure 2A, the occurrence of typical methacrylate peaks at 5.2–5.7 ppm testified the successful chemical modification to obtain the GelMA by the anhydride reaction. Figure 2B showed the similar morphology and the size of inner porous for these GelMA-CS and GelMA-CS@KGN composite hydrogels, which could satisfy the requirement of the pore size that allowed the cell infiltration, nutrition transfer, metabolic waste discharge, and other substance exchange. Taking consideration of the unchanged network architectures, we hypothesized the introduction of electrostatic interaction can significantly improve the drug release kinetics and encapsulated KGN drugs could also be enabled for the sustained release from the hydrogels, thereby conducting a slow release to maintain the sufficient drug concentration *in situ* for a long time to promote cartilage tissue regeneration.

Mechanical property is another important aspect for assessing the tissue engineered scaffold for cartilage repair. It is better for the mechanical parameters to be able to match the native cartilage. Therefore, we first carried out the rheological experiments in Figure 2C. The storage modulus (G′) surpassing the loss modulus (G″) within the frequency range revealed the hydrogel formation regardless of the addition of KGN molecules. The compressive strength of hydrogel scaffold is also assessed in Figure 2D. Both the GelMA-CS and GelMA-CS@KGN composite hydrogels exhibited the similar compressive strength to that of native cartilage, which was attributed to the rigid CS backbone and the dense chain entanglement network within the hydrogels. It was mentioned that the higher strength of GelMA-CS@KGN than GelMA-CS may ascribe to the ordered and evenly distributed drugs as well as the optimized electrostatic interactions among the polymeric network and small molecules, which also reflected the KGN loading did not affect the microarchitecture and mechanical performances of composite hydrogel scaffolds.

### Kartogenin Release Behavior of Composite Hydrogel *In Vitro*

**FIGURE 3:**
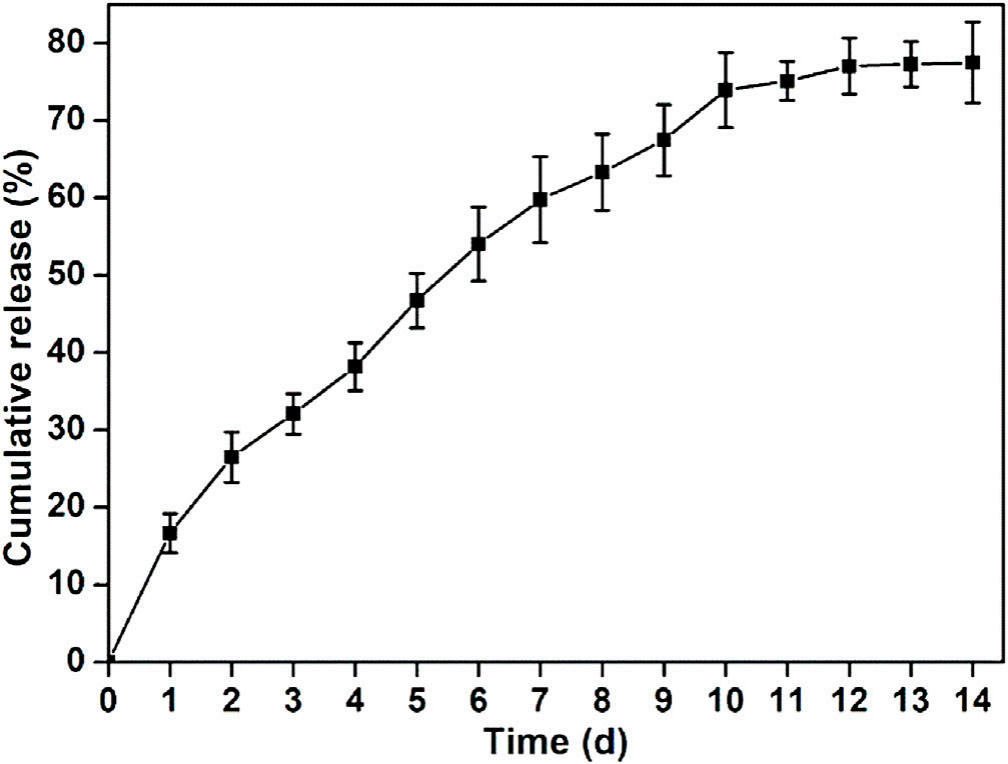
Release behavior of KGN from GelMA-CS@KGN composite hydrogel.

Although many traditional regulatory factors (e.g., TGF-β) can promote the cartilage repair, the short half-life and instability are the fatal defect because they always require at least 3 months achieving the reconstruction of damaged cartilage (Robson et al., 1995; Yang et al., 2001; Rey-Rico et al., 2015). In comparison, KGN is stable at room temperature and can effectively induce BMSCs into chondrocytes for a long term. The obvious superiorities in comparison to protein growth factors made KGN to be a potential chondrogenesis promoter in cartilage regeneration (Kang et al., 2017; Wang et al., 2019; Dehghan-Baniani et al., 2022). Moreover, several studies demonstrated that KGN possessed excellent biocompatibility without the toxicity on many cells even with a high concentration of 100 μM (Johnson et al., 2012). In this study, this concentration of KGN was selected and the release profile from the GelMA-CS@KGN hydrogel was detected in PBS solutions using UV−vis spectrophotometry with the absorption peak of 278.4 nm. In addition, because the absorption peak of KGN was at 278.4 nm, UV irradiation (365 nm) can not affect the KGN release for its therapeutic efficiency. A constant and sustained release of KGN is observed in Figure 3, which exhibited the cumulative release rate of KGN reached nearly 80% up to 14 days. It was mentioned that although a burst KGN release with 25% of cumulative release was displayed at the initial stage, this quick release could satisfy the high concentration demand in the early stage of defect areas for immunoregulation. This result revealed that the hydrogel scaffold got a well control release of KGN to simultaneously satisfy the initial high drug concentration demand and following long-term sustained release.

### Cell Viability and Proliferation

**FIGURE 4:**
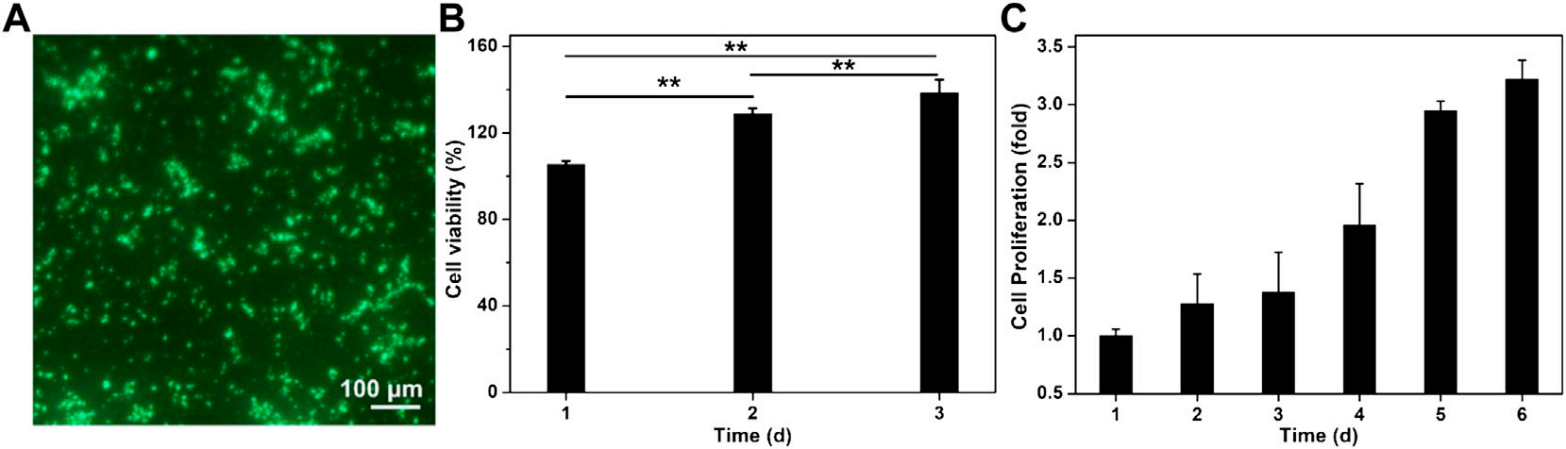
Cytotoxicity of GelMA-CS@KGN hydrogel *in vitro*. **(A)** Live/dead staining of BMSCs. Cells in green manifest living ones while cells in red manifest dead ones. **(B)** Cell viability. **(C)** Cell proliferation of GelMA-CS@KGN hydrogel after cultivating for various time (*n* = 3, **p < 0.01).

The polysaccharide CS and GelMA possessed excellent biocompatibility and favorable advantages in terms of cell attachment, growth, proliferation, and differentiation. Because KGN had no toxicity on cells even at a concentration of 100 μM, this GelMA-CS@KGN composite hydrogel was demonstrated to be an ideal engineered scaffold, which was testified using the live/dead assays. The cytotoxicity assay exhibited the gratifying result with green fluorescence after culturing GelMA-CS@KGN hydrogels into the BMSCs for 24 h in Figure 4A. Instead of cell apoptosis, extended incubation time led to the growing cell number with more than 120% of cell viability at day 2 and 3 (Figure 4B). Therefore, we further investigate if this hydrogel was able to support the cell growth and proliferation, a long-term proliferation profile of CCK-8 assay was carried out after treatment with GelMA-CS@KGN hydrogel for 6 days. Figure 4C showed that these composite hydrogels significantly promoted the cell growth and proliferation without any toxicity, and the proliferation rate slightly increased in the initial 3 days and rapidly arouse in the late 3 days, further revealing the excellent cytocompatibility of these cartilage scaffolds even if there were some KNG drugs leaking out from the GelMA-CS@KGN hydrogel. In addition, the degradation behavior of this composite hydrogel is similar to the traditional naturally derived hydrogel scaffolds, and their degradation mechanism can be attributed to the hydrolysis or enzymatic hydrolysis effects, which was affected by the degree of methyl acrylamide, the molecular weights of gelatin and CS, concentration, and the enzymatic degradation (Lin et al., 2021).

### RT-PCR Analysis

**FIGURE 5:**
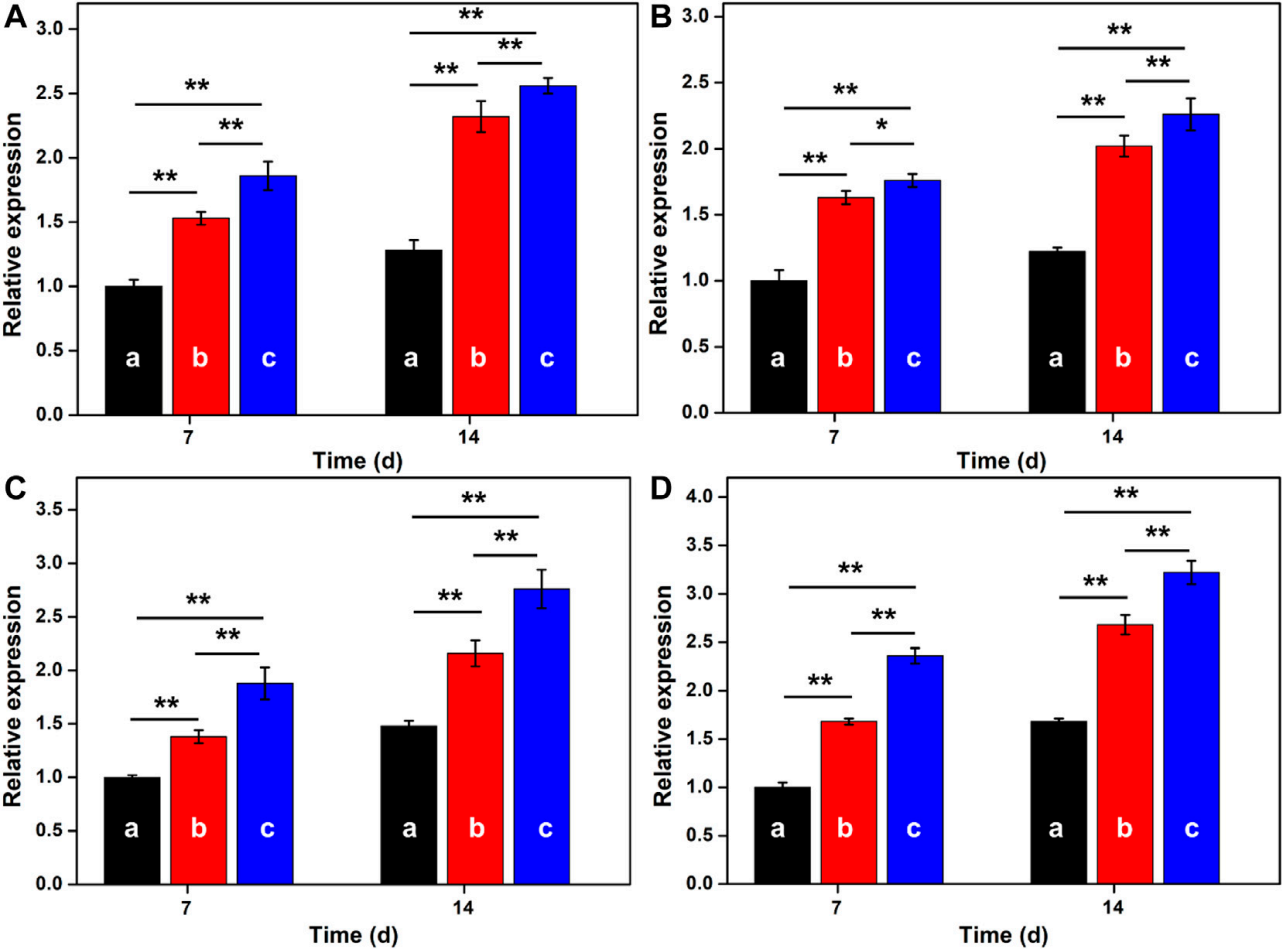
RT-PCR analysis. The mRNA expression of cartilage-specific marker genes of **(A)** ACAN, **(B)** COL-2, **(C)** PRG4, and **(D)** SOX-9 for **(A)** GelMA, **(B)** GelMA-CS, and **(C)** GelMA-CS@KGN (100 μg/ml) scaffolds determined by RT-PCR tests on days 7 and 14 (*n* = 3, *p < 0.05, and **p < 0.01).

Ideal cartilage repair materials should enhance the chondrogenic differentiation of BMSCs. To reveal the effect of hydrogel scaffolds on the chondrogenic differentiation *in vitro*, the expression of cartilage-specific genes like ACAN, COL-2, PRG4, and SOX-9 are investigated in Figure 5. Compared with the traditional GelMA hydrogel, real-time PCR showed that GelMA-CS and GelMA-CS@KGN (100 μg/ml) hydrogel scaffolds exhibited higher expression of cartilage-specific genes. It was mentioned that the improved mechanical property of GelMA-CS may contribute to the favorable cartilage differentiation ability *in vitro*. In addition, on account of the sustainable release of KGN drug, the mRNA expression levels of cartilaginous markers of ACAN, COL-2, PRG4, and SOX-9 were significantly upregulated in the GelMA-CS@KGN (100 μg/ml) group after incubation for 7 and 14 days (*n* = 3, *p* < 0.05), suggesting that the KGN agents could effectively promote chondrogenic differentiation of the BMSCs *in vitro* for a long term.

### Extracellular Matrix Deposition on Hydrogel Scaffolds

**FIGURE 6:**
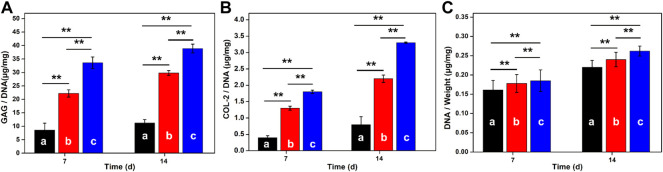
GAG production, COL-2 proteins and DNA contents in hydrogels after culturing for 7 and 14 days **(A)** GAG production, **(B)** COL-2 deposition, and **(C)** DNA contents in various hydrogels scaffolds of **(A)** GelMA, **(B)** GelMA-CS, and **(C)** GelMA-CS@KGN by the BMSCs on 7 and 14 days (*n* = 3, **p < 0.01).

ELISA analysis was used to quantitatively evaluate the extracellular protein produced by the MSCs in hydrogels in Figure 6. Along with the extension of time, DNA contents were continuously increased for these three groups. Wherein, the average DNA content in GelMA-CS@KGN was higher than the other groups within 2 weeks. Similarly, GAG and COL-2 contents in GelMA-CS@KGN hydrogels also increased over time, and reached their highest levels after 14 days that was more than other groups at any time points. To the best of our knowledge, GAG was an important ECM component in articular cartilage, and its production was directly related to the level of chondrogenesis. Thereafter, these results suggested that the KGN release from the hydrogel scaffold could significantly promote the chondrogenic differentiation of BMSCs.

## Conclusion

In summary, we developed a KGN-loaded GelMA-CS composite hydrogel with the long-term drug release. On account of the good compatibility, suitable porous architectures and favorable mechanical strength, this GelMA-CS@KGN hydrogel scaffold could promote the cell activity, growth, proliferation, and differentiation to favor the cartilage regeneration. Cytotoxicity assay indicated the good cell proliferation, while the sustained KGN release could significantly promote the cartilaginous differentiation *in situ*. The RT-PCR test also verified that the hydrogel scaffolds could enhance the expression of cartilage-specific genes as well as matrix deposition of the increased level of GAG, capable of facilitating cartilage tissue regeneration. Importantly, this composite hydrogel system could overcome the limitations of protein growth factor-based scaffolds and reach a stable chondrogenesis-inducing status for a long repair time. We believe this study demonstrated it may serve as an appropriate strategy for cartilage defect repair by locally delivering the stable chondrogenesis KGN promoter, which will exhibit great potential for clinical cartilage regeneration.

## Data Availability

The raw data supporting the conclusion of this article will be made available by the authors, without undue reservation.
